# Organic molecule functionalized lead sulfide hybrid system for energy storage and field dependent polarization performances

**DOI:** 10.1038/s41598-022-23909-z

**Published:** 2022-11-11

**Authors:** Sarit K. Ghosh, Ibrahim Waziri, Maolin Bo, Harishchandra Singh, Rafique Ul Islam, Kaushik Mallick

**Affiliations:** 1 Department of Chemical Sciences, University of Johannesburg, P.O. Box: 524, Auckland Park, 2006 South Africa; 2 Key Laboratory of Extraordinary Bond Engineering and Advanced Materials Technology (EBEAM) of Chongqing, Yangtze Normal University, Chongqing, 408100 China; 3 Nano and Molecular Systems Research Unit, University of Oulu, 90014 Oulu, Finland; 4 Department of Chemistry, School of Physical Sciences, Mahatma Gandhi Central University, Motihari, 845401 India

**Keywords:** Energy science and technology, Materials science, Nanoscience and technology

## Abstract

A wet chemical route is reported for synthesising organic molecule stabilized lead sulfide nanoparticles. The dielectric capacitance, energy storage performances and field-driven polarization of the organic–inorganic hybrid system are investigated in the form of a device under varying temperature and frequency conditions. The structural analysis confirmed the formation of the monoclinic phase of lead sulfide within the organic network. The band structure of lead sulfide was obtained by density functional theory calculation that supported the semiconductor nature of the material with a direct band gap of 2.27 eV. The dielectric performance of the lead sulfide originated due to the dipolar and the space charge polarization. The energy storage ability of the material was investigated under DC-bias conditions, and the device exhibited the power density values 30 W/g and 340 W/g at 100 Hz and 10 kHz, respectively. The electric field-induced polarization study exhibited a fatigue-free behaviour of the device for 10^3^ cycles with a stable dielectric strength. The study revealed that the lead sulfide-based system has potential in energy storage applications.

## Introduction

Semiconductor nanoparticles have attracted much attention because of their interesting electrical and optical properties, originating from quantum confinement effect^1^. Among the various semiconductor nanomaterials, metal sulphides have received much attention due to their appropriate electronic band gap and bandposition^2^. So far as application point of view, metal sulfides based devices displayed efficient performances in several areas including fuel cell^3^, solar cell^4^, light-emitting diode^5^, gas sensor^6^, battery^7^, supercapacitor^8^, thermoelectric^9^, dielectric^10^ and memory^11^ applications.The performance of the devices predominantly depends on the nano architecture of the metal sulfides.

In this context, lead sulfide, a binary (IV–VI) semiconductor material, with moderately small bandgap and large exciton Bohr radius (18 nm), stand out as promising material that has been successfully used for different applications such as infrared sensors^12^, solar cells^13^, light-emitting diodes^14^, lasers^15^ and biological imaging^16^ due to its controllable size with variety of morphologies. Different morphologies, such as rod and cube^17^, spherical and dendritic^18^, wire^19^, and star^20^ shaped lead sulfide has been reported, at nanometer to micrometer scale, by several synthetic methods, such as, microwave, hydrothermal, solvothermal, and chemical or thermal decomposition, under different reaction conditions (precursor varities, temperature, time, solvent and stabillizer)^17,21,22^.

Nanocrystalline lead sulfide also exhibited the catalytic performance for the synthesis of amidoalkyl-naphthols under solvent-free conditions^23^ and for the chemical reduction of *p*-nitroaniline^24^. The narrow bandgap, high electron mobility and excellent chemical stability enable the lead sulfide as a promising photocatalytic material^25^. The lead sulfide nanoparticle initiated photocatalytic degradation of bromothymol blue has been reported due to electron–hole pair generation mechanism^26^.

Employing the band gap engineering, using different ligands, the electrical property of lead sulfide based field-effect transistor showed the transformation from ambipolar to strong n-type behaviour^27^. The colloidal lead sulfide quantum dots also contribute to the improvement of solar cell efficiencies through better carrier extraction^28^. The dopped lead sulfide nanocrystal experienced an improvement of optical properties due to charge injection^29^. Optical properties of lead sulfide, based on theoretical calculation, exhibited good reflection and absorption for ultraviolet electromagnetic waves, suggested a potential candidate for photoconductive devices in ultraviolet range^30^. Lead sulfide exhibited immense potential in microelectronics application. The lead sulfide nanocrystal, with cubic symmetry, exhibited low dielectric constant and dielectric loss over a wide range of temperature and frequency conditions^31^. Nanoparticles of lead sulfide grown within the pores of polyvinyl alcohol matrix exhibited the moderate value of dielectric constant at higher frequencies^32^. It has been reported that lead sulfide nanoparticles, doped with strontium^33^ or cerium^34^, displayed an improved dielectric and electrical characteristics, compared to the pure lead sulfide. Two dimensional cubic phase lead sulphide nanosheets, synthesized using solid state reaction method, exhibited high values of dielectric constant and have potential as capacitive storage device^35^.

Lead sulfide with cubic structure is common in literature, Table 1, however the formation of monoclinic lead sulfide is relatively rare^36–38^. In this study, a complexation mediated wet chemical route was applied for the synthesis of fluoroaniline stabilized monoclinic lead sulfide nanoparticles. The dielectric capacitance, energy density, AC-conductivity and field driven polarization performances of the synthesized material was investigated in form of a device under varying temperature and frequency conditions. A comparative study was also performed on the device based on aniline stabilized lead sulfide system. A mechanism based on the performances of the two devices is also discussed in this manuscript.

**Table 1:** The synthesis methodology, structure and physical properties of some of the reported lead sulfide based system.

Material type	Synthesis method	Crystal structure	Electrical and physical properties	References
PbS nanocrystal	Solvothermal route	Cubic	Solar energy storage	^55^
Nanocrystal and nanocomposite of PbS	Ligand exchange and chemical synthesis route	–	Photoluminescent property and photovoltaic application	^56,57^
PbS nanocrystal	ligands mediated synthesis and chemical route	Cubic	Photovoltaic and photoluminescent property	^58,59^
PbS nanoplatelets	Chemical route	Orthorhombic	Morphology, band structure	^42^
Nanocomposite of PbS-graphene	Hydrothermal	Cubic	Gas sensor	^60^
PbS nanosheet	Colloidal synthesis route	Orthorhombic	Photoconductivity, optical absorption, band structure	^61^
PbS nanocrystal	Ligand based complexation	Cubic	ε′ ~ 13.4	^31^
Thin film of PbS	Chemical bath deposition	Cubic	ε′ ~ 155–265	^32^
Sr-doped PbS	Microwave route	Cubic	ε′ ~ 25–57, AC-conductivity	^33^
Ce-doped PbS	Chemical route	Cubic	ε′ ~ 17.5–25, DC-conductivity	^34^
Aniline and 4-fluoroaniline stabilized PbS nanocrystal	Ligand based complexation	Monoclinic	ε′ ~ 376 (ALS) ε′ ~ 800 (FALS) @ 100 Hz and 30 °C, AC-conductivity, energy and power density, polarization loop	Present work

## Result and discussion

**Figure 1:**
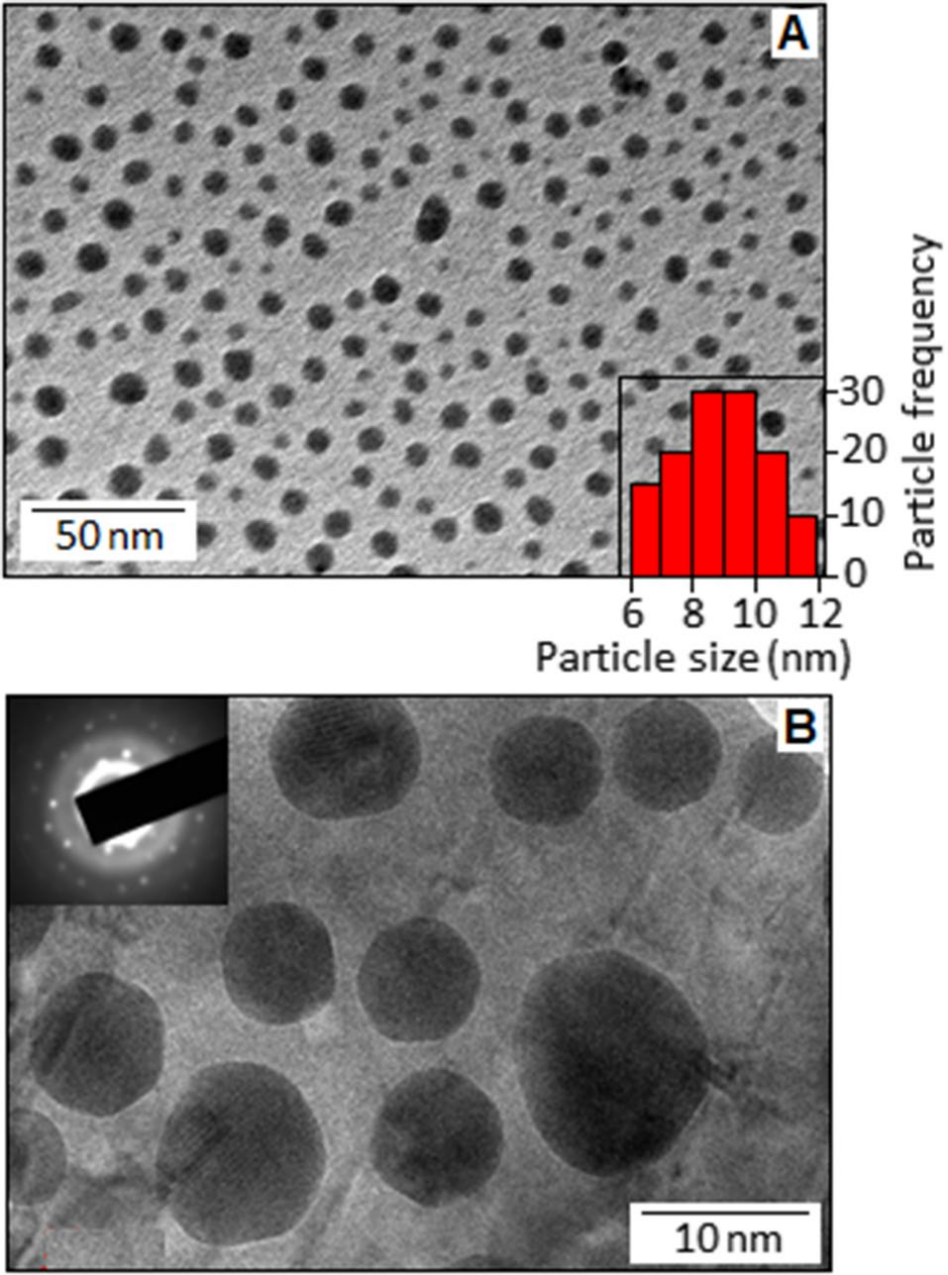
The transmission electron microscope images of the fluoroaniline stabilized lead sulfide nanoparticles with different magnifications (**A**, **B**). The inset image in (**A**) represent the size distribution of the particles and the inset image in (**B**) is the selected area electron diffraction pattern of lead sulfide.

A complexation mediated route was applied for the preparation of lead sulfide nanoparticles, where 4-fluoroaniline was served the role of a ligand and also as a stabilizer of the particles. The detail NMR characterization of the ligand and products are available in the supporting information (Table S1 and Figs. S1, S2). The TEM images, Fig. 1A,B, with different magnifications, of the fluoroaniline stabilized lead sulfide displayed the spherical shaped lead sulfide particles (dark spots) within the size range of 8–12 nm, presented using a histogram, Fig. 1A, inset. The selected area electron diffraction (SAED) pattern of lead sulfide displayed in Fig. 1B, inset, obtained from the groups of particles.

**Figure 2:**
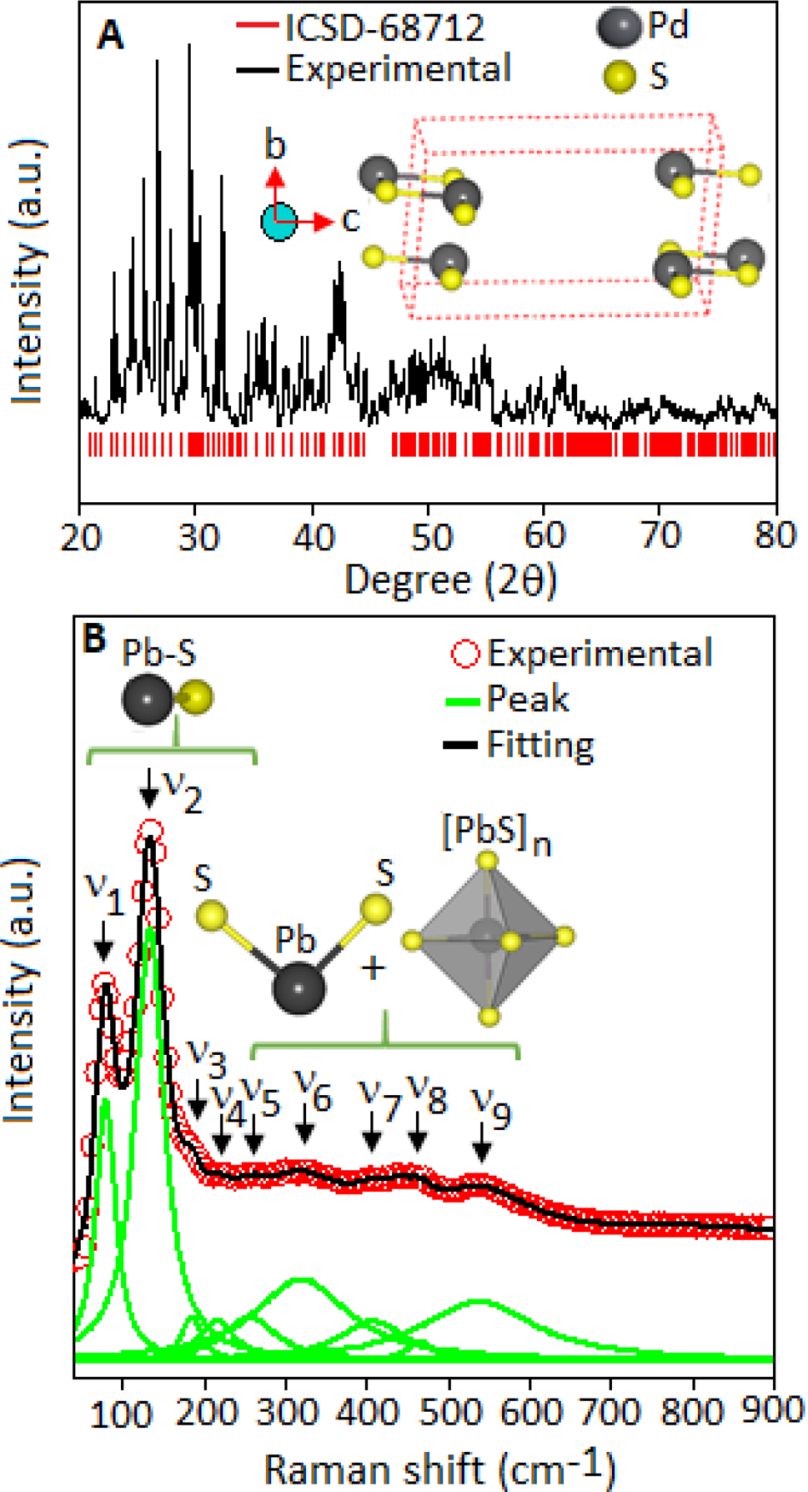
(**A**) X-ray diffraction pattern of lead sulfide (black line) was measured within the range of 20–80°. The diffraction pattern was indexed according to ICSD: 68712 (red colour bar) of the monoclinic structure. The inset figure shows the unit cell of lead sulfide projected along a-axis. (**B**) The deconvoluted Raman spectrum (green colour), within the range from 40 to 900 cm^−1^, with Raman active vibrational modes (ν_1_–ν_9_).

The X-Ray diffraction pattern of lead sulfide nanoparticles was recorded within the range (2θ) from 20° to 80°, Fig. 2A. The overall diffraction pattern was matched according to the ICSD: 68712, which belongs to the monoclinic phase of lead sulfide with the space group of P1. The monoclinic structure, with the lattice constant values, a = 6.00 Å, b = 6.02 Å, c = 22.43 Å and α = γ = 90°, β = 95.4°, accompanied with a large unit cell volume of 808.54 Å^3^. Figure 2A, inset, represents the three dimensional unit cell structure of lead sulfide, elongated along the c-axis. In lead sulfide, with the atomic arrangement of lead and sulphur along the 8-asymmetrical lattice sites in the structure^36–38^. The details positional coordinates of lead sulfide system is mentioned in Table S2, supporting information.

The local structure of lead sulfide (PbS) nanoparticles was investigated using Raman spectroscopy technique. The Raman spectrum (Fig. 2B), was deconvoluted (green colour peaks) into nine Raman active vibrational modes, from ν_1_ to ν_9_, within the range from 40 to 900 cm^−1^. The spectrum consists of strong intensity vibrational modes (ν_1_ and ν_2_), diffuse modes (ν_3_, ν_4_, ν_5_ and ν_8_) and broad intensity modes (ν_6_, ν_7_ and ν_9_). Raman active modes positioned at ν_1_ ~ 81 cm^−1^ and ν_2_ ~ 137 cm^−1^ along with a shoulder like feature at ν_3_ ~ 184 cm^−1^, originated from the longitudinal optical phonon vibration of Pb–S phase^39^. The diffuse modes ν_4_ ~ 216 cm^−1^ and ν_5_ ~ 254 cm^−1^ are associated to the symmetrical vibration of nonlinear S-Pb–S chain and the radial vibration of the of (PbS)_n_ cluster, (*n* = *1–9*), respectively, in the monoclinic structure^40^. The broad peak at ν_6_ ~ 328 cm^−1^, combined with smaller peaks at ν_7_  ~ 410 cm^−1^ and at ν_8_  ~ 470 cm^−1^, related to the various stretching modes of S–S bond in the structure^41^. The vibrational mode observed at ν_9_  ~ 546 cm^−1^ correspond to the phononic replicas of Pb–S phase^39^.

**Figure 3:**
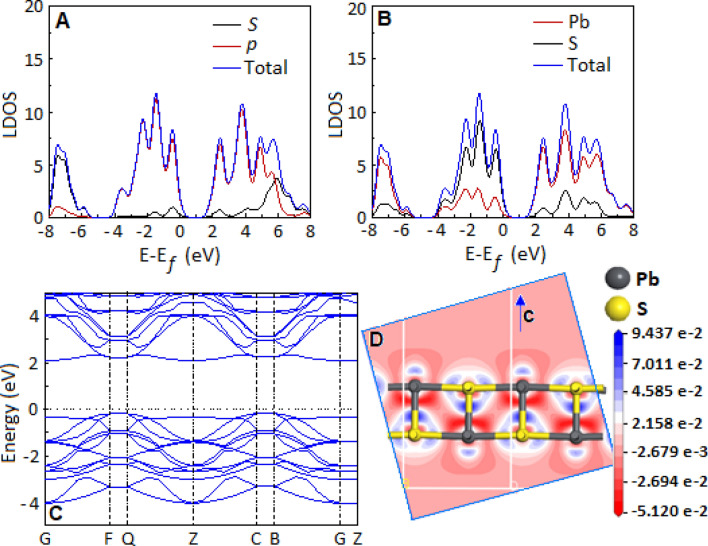
The distribution of local density of states of the lead sulfide. (**A**) The *s* and *p* orbital density of states and (**B**) the atomic, Pb and S, density of states. (**C**) Band structure of lead sulfide. (**D**) Deformation charge density of lead sulfide. (The Cambridge Sequential Total Energy Package, CASTEP, was used to optimize the geometry and calculating local charge and electronic properties, Supplementry information).

Figure 3A,B show the distribution of local density of states (LDOS) of lead sulfide. The structure illustrated the *s* and *p*-orbital contribution in the density of states (Fig. 3A) and the atomic density of states of Pb and S (Fig. 3B). The effective distribution of the states concluded that the valance band edge has *p-*orbital like character and typically localized with the S-atom, whereas the Pb-atom contribute more towards conduction band^42^. The band structure of lead sulfide was obtained by density functional theory calculation (Fig. 3C), that supported the semiconductor nature of the material with a direct band gap of 2.27 eV. Figure 3D represents the deformation charge density of the lead sulfide structure. The blue part represents the gain of electrons (electron population), and the red part symbolizes the loss of electrons (electron reduction).

**Figure 4:**
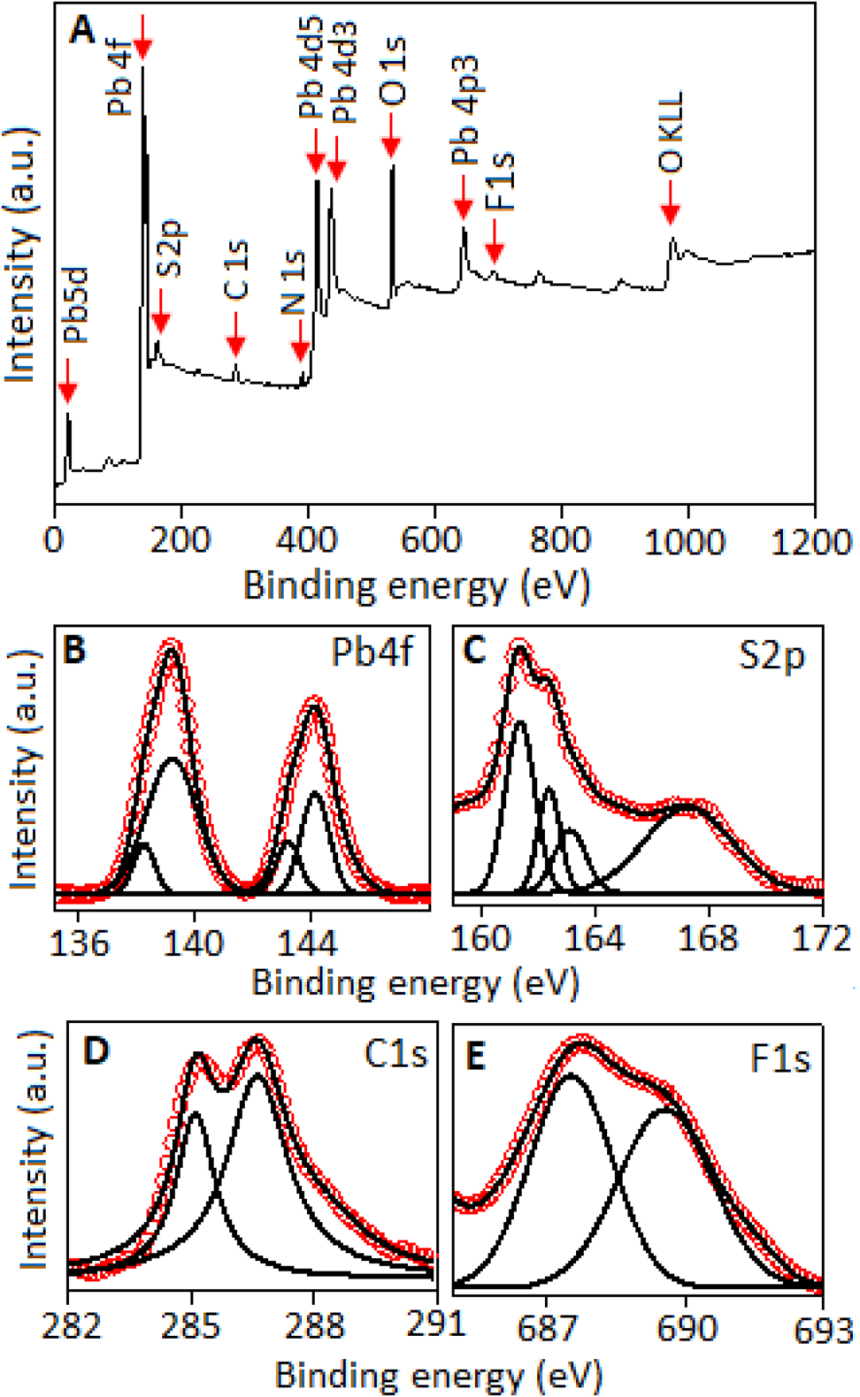
(**A**) The XPS survey spectrum of fluoroaniline stabilized lead sulfide. High-resolution XPS spectrum of (**B**) Pb 4f, (**C**) S 2p, (**D**) C1s and (**E**) F1s.

The qualitative analysis of the chemical states of the fluoroaniline stabilized lead sulfide nanocrystal was explored by X-ray photoelectron spectroscopy (XPS) technique. The survey spectrum (Fig. 4A), exhibited the presence of elemental peaks of C, N, O, F, S and Pb. The N (1 s) and O (1 s) peaks are positioned at 399.5 and 529.8 eV, respectively. The carbon, nitrogen and fluorine signals are originated from the organic matrix (fluoroaniline) and the O (1 s) signal is developed due to the surface oxidation of the sample. The binding energy peaks originated from the Pb 5d_5*/*2_, Pb 4f, Pb 4d_5*/*2_, Pb 4d_3*/*2_, and Pb 4p_3*/*2_ are also visible in the survey spectrum^43^. The high resolution Pb 4f spectrum (Fig. 4B) shows spin–orbit splitting of Pb (4f_7/2_) and Pb (4f_5/2_) with the peaks positioned at 139.1 eV and 143.8 eV, respectively, associated with the lead sulfide component^44^.

The deconvoluted of Pb 4f doublet peaks also display two additional small peaks positioned at 138.1 eV and 143.1 eV, originated from the surface oxidation of lead sulfide with the formation of lead sulfite (PbSO_3_) like species, under the exposure of aerial oxygen^44^. The high resolution S 2p core level spectrum, after deconvolution, Fig. 4C shows two peaks located at 161.5 eV and 162.7 eV, correspond to S (2p_3//2_) and S (2p_1//2_), for the sulfide ion of lead sulfide^44^. The formation of lead sulfite species, due to the surface oxidation of lead sulfide, was also evidenced in the spectrum, with the binding energy values positioned at 163.3 eV and 167.7 eV, also reported in other lead sulfide systems^44^. The values of the binding energy were calibrated utilizing C 1 s peak at 285.1 eV, as the internal standard, associated with the carbon from the aromatic ring (Fig. 4D). Other C 1 s peak with the binding energy value of 286.6 eV related to -C-F interaction form fluoroaniline^45^. Further, after deconvolution of the F1s spectra (Fig. 4E), two peaks are developed. The peak with the binding energy value of 689.6 eV is originated from the fluorine in fluoroaniline molecule and the other peak at 687.5 eV associated with lead-fluorine interaction from the neighbouring fluoroaniline molecule.

**Figure 5:**
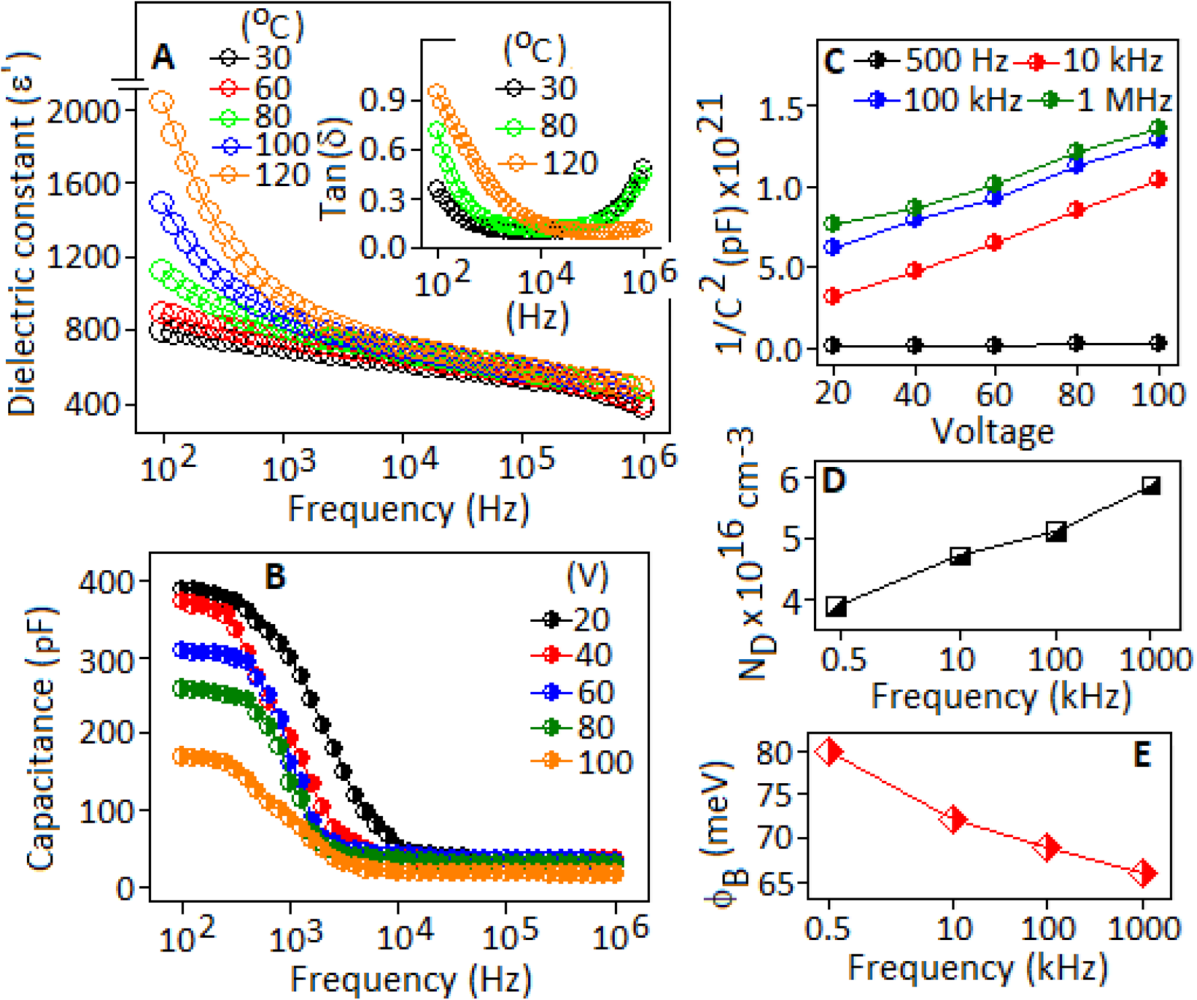
(**A**) Dielectric constant (ε′) of FALS based device measured at 30, 60, 80, 100 and 120 °C under the frequency range from 100 Hz to 1 MHz. The variation of tan δ curves for selected temperatures, inset. (**B**) Graphical representation of capacitance as a function of frequency under applied voltage conditions. (**C**) 1/C^2^–V plot under selected frequency conditions. (**D**) and (**C**) represent the variation of carrier concentration (N_D_) and barrier potential (ϕ_B_) as a function of frequency, for FALS based device.

**Scheme 1:**
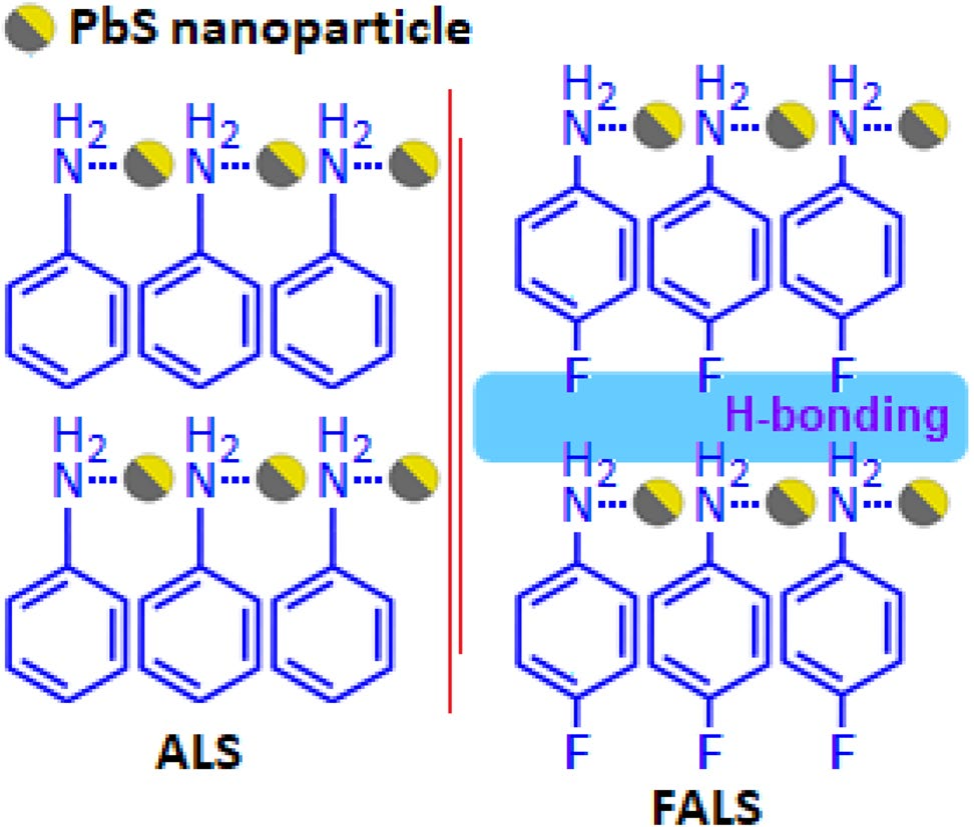
The schematic diagram of aniline and fluoroaniline stabilized lead sulfide nanoparticles. Hydrogen bond formation in fluoroaniline stabilized lead sulfide system.

Figure 5A shows the temperature (from 30 to 120 °C) and frequency (from 100 Hz to 1 MHz) dependent dielectric constant (εʹ) of FALS based device. At 30 °C, the value of dielectric constant (εʹ) ~ 800 was achieved under the frequency condition of 100 Hz. With increasing the frequency, the dielectric constant decreased gradually and attained a stable value of εʹ ~ 360 at 1 MHz. At 120 °C, a substantial rise of the value of dielectric constant (εʹ) ~ 2000 was observed at the frequency of 100 Hz, which was also decreased gradually towards higher frequencies. The capacitive behaviour of the lead sulfide was associated to the dipolar type of polarization. The dipoles are formed due to the separation of bound charges in the form of Pb^2+^ cation and S^2−^ anion, under the applied electric field. The structural analysis showed the deformation of charge density in the lead sulfide system and the charge transfer is directed towards the high electronegative sulphur atom. This kind of charge separation caused the formation of dipole moments. At lower frequency region, the induced dipoles are effectively followed with the applied field and attained a maximum value of dielectric constant. With increasing frequency, the dipoles are not able to respond with the field direction, which caused the decrease of dielectric constant values. The capacitive behaviour of the FALS based device was associated with the dipolar type of polarization (originated from the lead sulfide and highly polarised fluoroaniline molecule) and the space charge or interfacial polarization. The hydrogen atom of -NH_2_ group in fluoroaniline forms intermolecular hydrogen bond with the neighbouring fluorine of fluoroaniline, resulted high negative charge density (Scheme 1). This interface creates space charge within the system, which prompted further increase of dielectric constant value. In addition to that, the defect site of the nanoparticles and the organic–inorganic interface create the space charge within the system^10,46,47^. The space charge polarization is predominant towards the lower frequency range. The temperature effect on the dielectric constant of the device was associated with the loss factor of the material, as evidenced from the tan δ curve (Fig. 5A, inset). The rise in the tan δ curve with increasing the temperature implies that the space charges are thermally activated under the longer response time.

The dielectric polarization of the device was further analysed in term of capacitance as a function of frequency, under a series of voltage conditions (from 20 to 100 V) (Fig. 5B). From the figure it is evident that at lower frequency and lower voltage the change of capacitance is more favourable. At low voltage condition the polarization of the dipoles become facile. With increasing external voltage, the dipoles of the lead sulfide are aligned along the field direction with a restriction of the reversible polarization that resulted the decrease of capacitance value. Figure 5C exhibited the graphical representation of the inverse of capacitance (1/C^2^) as a function of voltage (V) under the frequency condition of 500 Hz, 10 kHz, 100 kHz and 1 MHz. The slope of the plots suggests that the negative charge carriers are dominant in the lead sulfide system^27,28^. The variation of carrier density (N_D_) and barrier potential (ϕ_B_) with respect to the frequency is plotted in the Fig. 5D,E, respectively. The increasing value of carrier density with frequency originated from the localized electronic state of the material with decreasing the value of barrier potential^48,49^. The dipolar contribution, originated from the bound charges of lead-sulfide system, is responsible for the localized deformation of electronic structure under applied frequency conditions.

**Figure 6:**
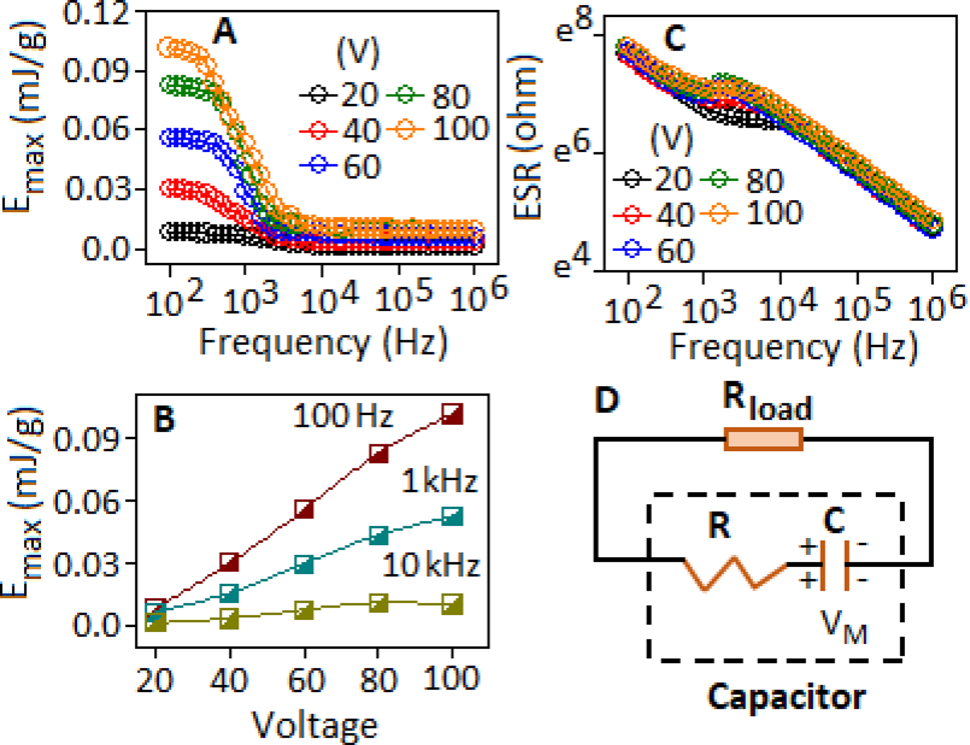
(**A**) Variation of energy density (E_max_) with respect to frequency, under DC-voltage, (**B**) energy density (E_max_) as a function of voltages under different frequency condition, (**C**) equivalent series resistance (ESR) as a function of frequency under different voltage condition (in log–log scale), (**D**) equivalent circuit representation for the energy and power transfer model at a certain voltage (V_M_), for the FALS based device.

The maximum energy storage density (*E*_*max*_) of the FALS based device was calculated under different DC-bias condition using the equation, ${E}_{max}=\frac{1}{2} C{V}_{M}^{2}$ , where *C* represent the DC capacitance and V_M_ is the applied voltage. The E_max_ value of ~ 0.10 mJ/g was achieved at 100 V (under 100 Hz frequency condition) and the *E*_*max*_ values decreased with increasing frequency (Fig. 6A). The variation of E_max_ is more prominent towards lower frequencies (below 1 kHz) under high voltage condition (Fig. 6B). The equivalent series resistance (ESR) of the device was extracted from AC-impedance measurement, which exposed an insignificant variation under different bias conditions (Fig. 6C) (in log–log scale). The circuit representation of the device Fig. 6D), can be sketched by assuming that the stored energy was discharged through a load resistance ${(R}_{load})$, connected in parallel with a capacitor (*C*) and a resistor (*R*). The product of capacitance and resistance is the time constant (*t*) and the ratio of energy density to time constant (*RC*) gives the power density, which is a gravimetric power density that determine the minimum time required to discharge the stored energy of the device. The maximum power ${(P}_{max})$ delivered to the load resistance can be expressed as ${P}_{max}={E}_{max}/2RC$. The calculated RC values were obtained 1.5 ms and 16 μs with the gravimetric power density values of 30 W/g and 340 W/g, at 100 Hz and 10 kHz, respectively, for the lead sulfide based device. A table of comparison based on the previously published results on gravimetric power density values for other organic–inorganic hybrid systems is mentioned in the supporting information, Table S3, as a ready reference.

**Figure 7:**
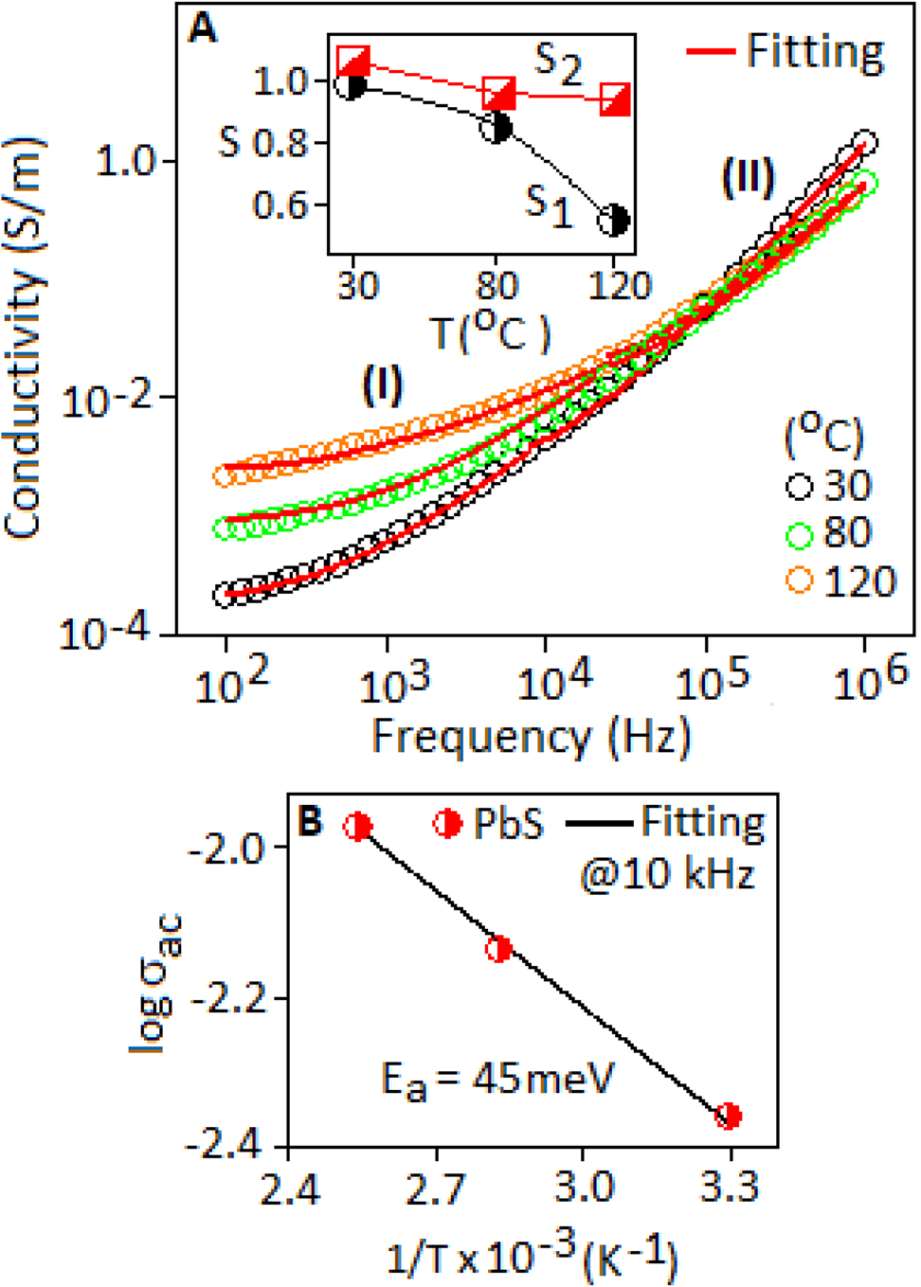
(**A**) AC-conductivity behaviour of the FALS based device at 30, 80 and 120 °C, measured under the frequency range from 100 Hz to 1 MHz (fitting was done according to the Jonscher’s power law, red line). The inset figure shows the variation of S_1_ and S_2_ parameters, extracted from the region (I) and (II), respectively. (**B**) Arrhenius plot of AC-conductivity as a function of inverse temperature (1/T), at 10 kHz.

The mobility of the charge carrier of the FALS based device was analysed in terms of AC-conductivity as a function of frequency for 30, 80 and 120 °C (Fig. 7A). Figure shows that the AC-conductivity is directly proportional to the applied frequency and also with the temperature. At 100 Hz, the conductivity values are 2.1 × 10^–4^ S.m^−1^ and 2.3 × 10^–3^ S.m^−1^ at 30 and 120 °C, respectively. The conductivity curves, for each temperature, has two regions, designated as region (I) and region (II), marked in the Fig. 7A. All the conductivity curves are finally merged towards the region (II). The region (I) exhibits a slow progress of conductivity with increasing frequency, on the other hand, the conductivity increases sharply with increasing the frequency, in the region (II). The conductivity behaviour was analysed in term of Jonscher’s universal power law,${\sigma }_{ac}= {\sigma }_{dc}+A\left(T\right){\omega }^{S},$ where, σ_dc_ is the DC-part of the conductivity, A is the temperature dependent coefficient of the system and $S$ is the temperature dependent parameter that determine the conduction mechanism of the charge carriers. In the low frequency region (I), the slow progress of conductivity with frequency, indicates few trapped charges are available for the conduction process, whereas the region (II) specify that large number of trapped charges are available in the conduction process. Several models have been proposed to study the conduction mechanism of the charge carriers^50^. It is reported that the polaron and bipolaron types of charge carriers are responsible for conduction mechanism in hybrid material^51^. The hybrid system of metal sulfides and metal halides demonstrated the formation of polaronic charge species that control the hopping and tunnelling type of transport mechanism^47,52^. The conductivity curves were fitted using Jonscher’s power law for the regions (I) and (II) (Fig. 7A, red line), and the extracted S_(I)_ and S_(II)_ parameters were plotted in Fig. 7A, inset. The values of S_(I)_ and S_(II)_ are decrease with increasing temperature, suggested the hopping conduction of polaron was involved in the lead sulfide based device system. The variation of AC-conductivity (${\sigma }_{AC}$) with temperature (1/T), described by Arrhenius equation, ${\sigma }_{AC}\left(T\right)={\sigma }_{0}\mathrm{exp}\left(-{E}_{a}/{K}_{B}T\right),$ ${\sigma }_{0}$ is pre-exponential factor and ${E}_{a}$ is activation energy, was measured at the frequency of 10 kHz. The E_a_ value was obtained ~ 45 meV, extracted from the linear fit of the equation (Fig. 7B). The small E_a_ value recommended the polaron mediated conduction within the system^53^.

**Figure 8:**
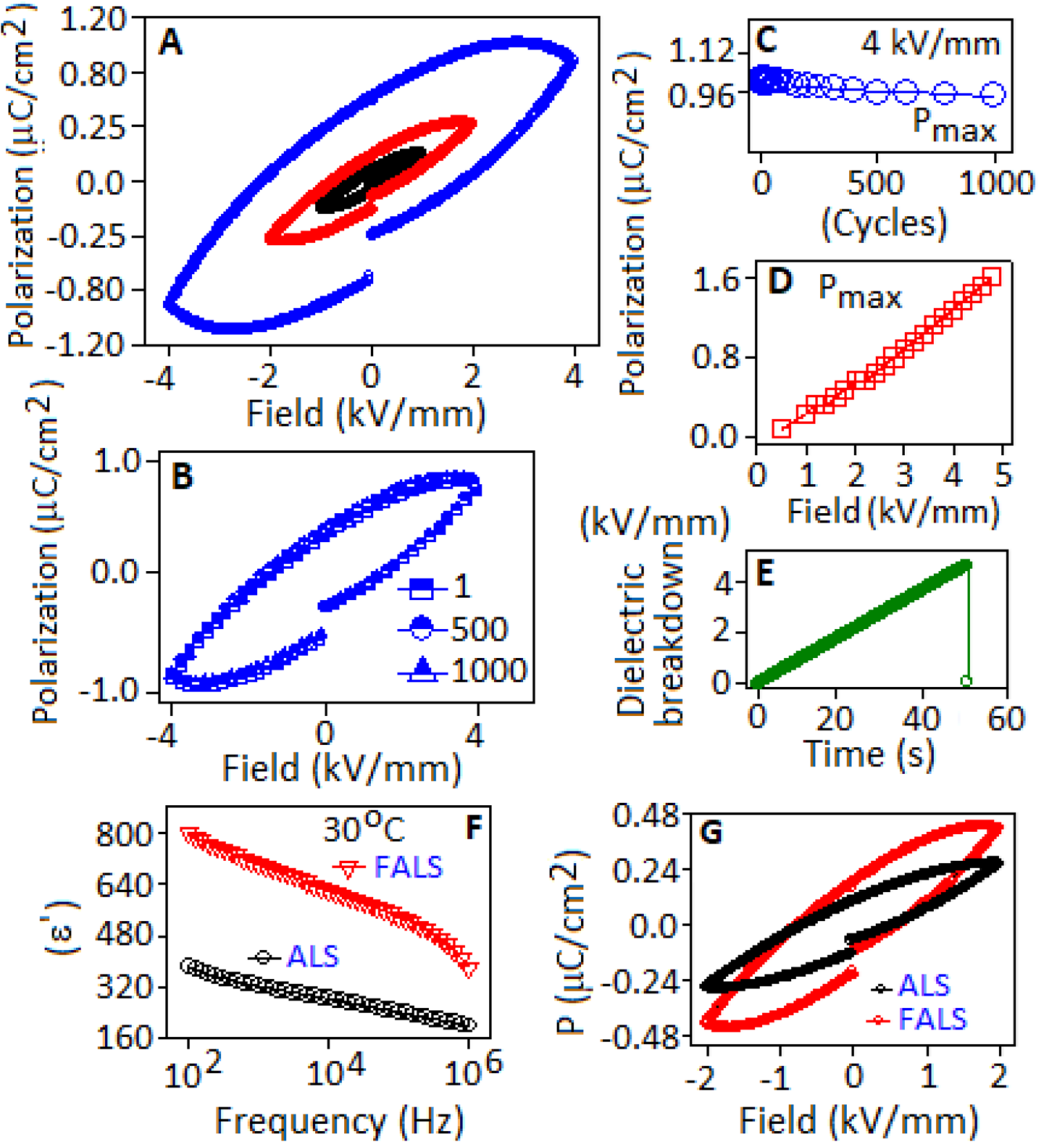
(**A**) Hysteresis loop, polarization vs electric field, of the FALS based device, measured at 100 Hz. (**B**) Hysteresis loop of the device under 4 kV/mm field condition, for 10^3^ cycles. (**C**) Endurance behaviour of the device (in terms of maximum polarization, P_max_) for 1000 cycles, at 4 kV/mm. (**D**) Increment of maximum polarization (P_max_) values with respect to the applied electric field. (**E**) Dielectric breakdown strength (kV/mm) of the device as a function of time, measured at a scan rate of 200 V/s. Variation of (**F**) dielectric constant (ε′) and (**G**) polarization vs electric field, hysteresis loop of ALS and FALS based device.

Figure 8A shows the polarization–electric field (P–E) hysteresis loop for the FALS based device, measured under 1, 2 and 4 kV/mm, field conditions. The dipolar type of polarizability of lead sulfide was responsible for the formation of hysteresis loop. The unsaturated pattern of the loops was associated with the space charge contribution in the system^54^. A steady polarization hysteresis loop was observed for the device at ± 4 kV/mm (Fig. 8B), and maintained the maximum polarization value within the range from 1.1 to 0.99 μC/cm^2^ for 10^3^ cycles (Fig. 8C). The polarizability of the covalent bond of the lead sulfide system was increased with increasing applied electric field and reached to the maximum polarization (P_max_) value of 1.6 μC/cm^2^ at 5 kV/mm (Fig. 8D). The dielectric breakdown strength of the device exhibited the tolerance up to 5.2 kV/mm of electric field, without any electrical breakdown and above that field the device experienced an electrical breakdown after 50 s (Fig. 8E).

A comparative study was performed between aniline and fluoroaniline stabilized lead sulfide based devices to investigate the role of fluorine. A similar synthesis method, like FALS, was applied for the preparation of aniline stabilized lead sulfide nanoparticles (ALS).

The aniline stabilized lead sulfide (ALS) and fluoroaniline stabilized lead sulfide (FALS) exhibited the dielectric constant values (ε′)  ~  376 and ~ 800, respectively, at 30 °C under 100 Hz frequency condition (Fig. 8F). The graphical representation of polarization as a function of electric field exhibited the hysteresis loop with the maximum polarization values 0.29 and 0.46 μC/cm^2^ for ALS and FALS based devices, respectively, at 2 kV/mm (Fig. 8G). High dielectric constant value of FALS was associated with the dipolar type of polarization and space charge polarization. In FALS, the dipolar type of polarization was created from the lead sulfide and polarised fluoroaniline molecule, whereas in ALS only the dipoles of lead sulfide are responsible for polarization.

The hydrogen atom of the –NH_2_ group forms intermolecular hydrogen bonding with the fluorine of FALS (Scheme 1) resulted high negative charge density. This interface procedures additional space charge in the system, which prompts further enhancement of the dielectric constant value in FALS based device. The increased area of the hysteresis loop and maximum polarization value of the FALS based device as compared with ALS based device were originated due to the above reasons.

Lead sulfide, with different size, shape and morphology, have been extensively investigated because of their unique optical and electronic properties, which endows the various potential applications. The electrical and physical properties of some reported lead sulfide based systems are mentioned in Table 1^31–34,42,55–61^.

## Conclusion

A complexation mediated wet chemical route was applied for the preparation of fluoroaniline stabilized monoclinic lead sulfide nanoparticles. The sizes of the synthesized particles are in the range of 8–12 nm, as evidenced from the microscopy analysis. The Raman spectrum analysis showed the different vibrational modes associated with lead sulfide. The X-ray photoelectron spectroscopy analysis confirmed the formation of lead (II) sulfide. The fluoroaniline stabilized lead sulfide based device exhibited the dielectric constant value of ~ 2000 at 120 °C at 100 Hz. The capacitive behaviour was associated with the dipolar type of polarization and the space charge polarization. The AC-conductivity values of the device were obtained 2.1 × 10^–4^ S m^−1^ and 2.3 × 10^–3^ S m^−1^ at 30 and 120 °C, respectively, at 100 Hz and the conductivity mechanism was followed by the hopping conduction of the polarons. The gravimetric power density values of 30 W/g and 340 W/g, at 100 Hz and 10 kHz, respectively, were achieved for the device. The device showed the maximum polarization value of 1.6 μC/cm^2^ at 5 kV/mm. A quasi-stable maximum polarization value in the range of 1.1–0.99 μC/cm^2^ was noted at 4 kV/mm, for 10^3^ cycles. Based on the above mentioned performances we could assume that the device has the potential as a pulse capacitor in microelectronic application.

## Experimental section

### Materials

Analytical grade chemicals (lead nitride*,* sodium sulfide and *4-*fluoroaniline) were used in this study without further purification.

### Synthesis of lead sulfide nanoparticles

In a typical experiment, 1.0 mL of *4-*fluoroaniline was diluted in 10 mL of methanol in a 50 mL conical flask. An aqueous solution of lead nitrate (10 mL) with the concentration of 0.1 mol dm^−3^ was added to the diluted *4-*fluoroaniline. A white precipitation of Pb(II)-fluoroaniline complex was formed immediately. To this precipitation, 5 mL of Na_2_S solution (0.1 mol dm^−3^) was added and instant change of colour of the precipitation in to black indicate the formation of fluoroaniline stabilized lead sulfide, FALS. The solid material was collected through filtration and dried under vacuum condition. The electrical properties of the synthesized materials were measured in the form of a device. The material characterization and device fabrication methods are available in the supporting information. The conditions set for computational analysis is also available in the supporting information.

## Supplementary Information

Supplementary Information.

## Data Availability

Data used for analyses in this manuscript are available from the corresponding author upon reasonable request.
