# Supplementary material for: Organic molecule functionalized lead sulfide hybrid system for energy storage and field dependent polarization performances
Source: Sci Rep. 2022 Nov 11;12:19280. doi: 10.1038/s41598-022-23909-z (PMC9652461; doi:10.1038/s41598-022-23909-z)
Supplement: Supplementary file 1 — Supplementary Information. [file 41598_2022_23909_MOESM1_ESM.docx]

**Supplementry information**

Organic molecule functionalized lead sulfide hybrid system for energy storage and field dependent polarization performances

Sarit K Ghosh^1^, Ibrahim Waziri^1^, Maolin Bo^2^, Harishchandra Singh^3^, Rafique Ul Islam^4^, Kaushik Mallick^1*^

^1^ Department of Chemical Sciences, University of Johannesburg, P.O. Box: 524, Auckland Park, 2006, South Africa.

^2^ Key Laboratory of Extraordinary Bond Engineering and Advanced Materials Technology (EBEAM) of Chongqing, Yangtze Normal University, Chongqing 408100, China.

^3^ Nano and Molecular Systems Research Unit, University of Oulu, FIN-90014, Finland.

^4^ Department of Chemistry, School of Physical Sciences, Mahatma Gandhi Central University, Motihari 845401, India.

E-mail: [kaushikm@uj.ac.za](mailto:kaushikm@uj.ac.za) (K Mallick)

**Material characterization:** The powder X-ray diffraction (XRD) property of the synthesized material was investigated using a Philips PANalytical X’pert diffractometer with Cu-K*α* radiation. Raman spectrum was recorded by Jobin-Yvon T64000 spectrometer using an argon ion laser source with an at excitation of 514.5 nm. The Nuclear magnetic resonance (NMR) spectra were recorded on a Bruker 500 MHz NMR spectrometer. Microscopic study was performed using JEOL (JEM-2100) analytical Transmission Electron Microscope (TEM). The dielectric performance was measured using HP 4284A LCR meter interfaced with a computer controlled EC1A environmental chamber. Electric field-dependent polarization study was performed using a Sawyer-Tower circuit and the sample was subjected to a triangular wave with a frequency of 100 Hz.

**Device fabrication:** The fluoroaniline stabilized ultrafine lead sulfide (FALS) based device was fabricated according to the following protocol. Initially, a slurry was made with the synthesized material using chloroform and deposited in the form of a film (thickness ~100 μm, sample weight ~5mg) on a copper plate. The copper plate was dried on a hot plate for 30 minute and the top gold electrode (area ~ 21 mm^2^) was printed above the film using physical vapour deposition technique (EMSCOPE SC 500). The electrical property of the fabricated device was measured under varying temperature and frequency condition. For a comparative study, ALS based device was also fabricated according to the above mentioned protocol. The ALS based device was only applied for the dielectric and the electric field induced polarization studies.

**Computational analysis of electronic band structure and density of state:** The energetics and electronic properties of lead sulfide nanoparticles was analyzed from first principles. The Cambridge Sequential Total Energy Package (CASTEP) software^1^ was used to optimize the geometry and also to calculate the local charge and electronic properties of material. The DFT calculation was performed using super-soft pseudo-potential and exchange correlation functions of the Perdew-Burke-Ernzerhof approximation^2^. To avoid interactions between repeated images, we have used a vacuum space of about 14 Å perpendicular to the plane of the lattice. Energy convergence criterion and the plane wave cut-off energy were set to 1×10^-6^ eV and 500 eV, respectively. The k*-*point mesh of the Brillouin zone was set to 8*×*8*×*2.

**Nuclear magnetic resonance (NMR) spectroscopy study:**

Nuclear magnetic resonance (NMR) spectroscopy analysis was performed for the 4-fluoroaniline (FA), Pb(II)-fluoroaniline (LFA) and fluoroaniline stabilized lead sulfide (FALS) using DMSO-d_6_ as a solvent (TMS as internal standard), under room temperature. The information regarding the formation of FALS can be obtained by predicting the chemical behavior of the proton of the FA and LFA. The molecular structure of FA with different chemical environment of the proton is displayed in figure S1. The proton NMR data and spectrum of FA are displayed in table S1 and figure S2 (A), respectively. The ^1^H NMR spectra of the complexation product, LFA, figure S2 (B), due to the reaction between FA and lead nitrate exhibited varying degree of shift of all the protons, table S1. The chemical shift observed is due to the decrease in the electron density on the nitrogen atom and the formation of strong bond between the nitrogen atom and Pb (II) ion. This interaction causes the amine protons and the aromatic proton, close to the amino group, to be deshielded and subsequently appeared in downfield region in the NMR spectrum. In addition, sigma (δ) effect is also responsible for the downfield shift of all the position of the protons in the LFA complex system. The formation of fluoroaniline stabilized lead sulfide (FALS) due to the addition of Na_2_S to Pb(II)-fluoroaniline, a further deshielding effect is observed and the protons appears in more downfield region in the NMR spectrum, figure S2 (C).

**Table S1:** ^1^H NMR data of the 4-fluoroaniline, Pb(II)-fluoroaniline and fluoroaniline stabilized lead sulfide (FALS).

| Sample | Chemical shifts (ppm) |
| --- | --- |
| 4-fluoroaniline (FA) | ^1^H NMR (500 MHz, DMSO-d_6_): δ = 6.85 (2H, t, *J* = 8.5 Hz, Ar-*H*),  6.58 (2H, m, *J* = 5.0 Hz, Ar-*H*), 4.91(2H, br, N*H*_2_) |
| Pb(II)-fluoroaniline (LFA) | ^1^H NMR (500 MHz, DMSO-d_6_): δ = 7.24 (2H, d, *J* = 8.0 Hz, Ar-*H*),  6.40 (2H, d, *J* = 8.5 Hz, Ar-*H*), 5.26 (2H, br, N*H*_2_) |
| FALS | ^1^H NMR = 7.01 (2H, d, *J* = 8.5 Hz, Ar-*H*), 6.56 (2H, d, *J* = 8.0 Hz, Ar-  *H*), 5.33 (2H, br, N*H*_2_) |

d = doublet, t = triplet, m = multiplet, and br = broad


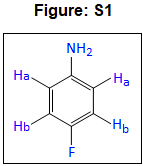


**Figure S1:** Molecular structures of the ligand showing the chemical environment of protons.


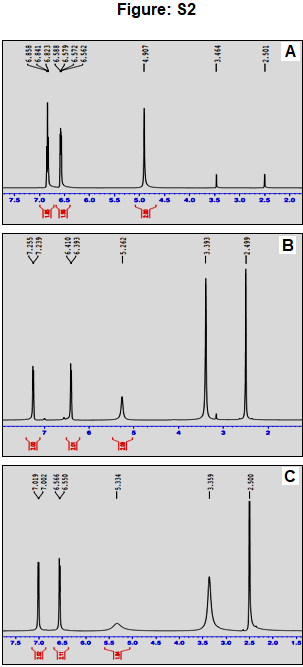


**Figure S2:** ^1^H NMR spectra of (A) FA, (B) LFA and (C) FALS.

**Table S2**: Positional coordinates of Pb and S atoms obtained after geometric optimization.

|  | x | y | z |
| --- | --- | --- | --- |
| Pb | 0.36308 | 0.91110 | 0.25753 |
| Pb | 0.81854 | 0.91110 | 0.13235 |
| Pb | 0.86308 | 0.41110 | 0.25753 |
| Pb | 0.31854 | 0.41110 | 0.13235 |
| S | 0.86146 | 0.91191 | 0.25308 |
| S | 0.32016 | 0.91191 | 0.13680 |
| S | 0.36146 | 0.41191 | 0.25308 |
| S | 0.82016 | 0.41191 | 0.13680 |

**Table S3**: Table of comparison on gravimetric power density values of other reported organic-inorganic hybrid systems.

| **Organic-inorganic hybrid materials** | **Device mechanism** | **Gravimetric power density** | **Ref.** |
| --- | --- | --- | --- |
| Hybrid perovskite-  lead halide and bismuth halide | Electrochemical pseudo-capacitor | 93.8 W/g and 58.7 W/g at 1.0 V/s scan | 3 |
| Tantalum oxide-ruthenium oxide hybrid capacitor | Solid state capacitor-  thick film | 19-259 W/g at 1 kHz | 4 |
| Hybrid composite PbO_2_/activated carbon | Electrochemical pseudo-capacitor | 433 W/kg | 5 |
| Hybrid nanocomposite NiFe_2_O­_4_ | Solid state capacitor-  bulk pellet | 9.91 W/g at 1 kHz condition | 6 |
| Nanocomposites  Nb_4_N_5_/rGO | Electrochemical pseudo-capacitor | 112 W/kg | 7 |
| Sodium ion hybrid pseudo-capacitor | Electrochemical pseudo-capacitor | 11-106 W/g | 8 |
| PEDOT-WO_3_-CeO_2_ nanowire | Electrochemical pseudo-capacitor | 801 W/g at 1.6 V condition | 9 |
| 4-fluoroaniline stabilized PbS (FALS) | Solid state capacitor-  thin film | 30 W/g @ 100 Hz and  340 W/g @ 10 kHz | Present work |

References:

1. S. J. Clark, M. D. Segall, C. J. Pickard, P. J. Hasnip, M. J. Probert, K. Refson, M. C. Payne, First principles methods using CASTEP, Zeitschrift fuer Kristallographie 220 (2005) 567-570 2. B. Hammer, L.B. Hansen, J.K. Nørskov, Improved adsorption energetics within density-functional theory using revised Perdew-Burke-Ernzerhof functionals, Phys. Rev. B 59 (1999) 7413

3. L. E. Oloore, M. A. Gondal, A. Popoola, I. K. Popoola, Surface capacitive charge storage in carbon nanodots-anchored hybrid halide perovskites, Carbon 173 (2021) 1048-1058.

4. T. Y. Chang, X. Wang, D. A. Evans, S. L. Roberson, J. P. Zheng, Characterization of tantalum oxide-ruthenium oxide hybrid capacitors, IEEE Trans. Ind. Electron. 51 (2004) 1313-1317.

5. W. Zhang, H. Lin, H. Kong, H. Lu, Z. Yang, T. Liu, High energy density PbO_2_/activated carbon asymmetric electrochemical capacitor based on lead dioxide electrode with three-dimensional porous titanium substrate, Int. J. Hydrog. Energy  39 (2014) 17153-17161.

6. R. Magisetty, P. Kumar, V. Kumar, A. Shukla, B. Kandasubramanian, R. Shunmugam, NiFe_2_O_4_/Poly(1,6-heptadiyne) nanocomposite energy-storage device for electrical and electronic applications, ACS Omega, 3 (2018) 15256-15266.

7. S. Li, T. Wang, Y. Huang, Z. Wei, G. Li, D. Ng, J. Lian, J. Qiu, Y. Zhao, X. Zhang, J. Ma, H. Li, Porous Nb_4_N_5_/rGO nanocomposite for ultrahigh-energy-density lithium-ion hybrid capacitor, ACS Appl. Mater. Interfaces, 11 (2019) 24114-24121.

8. Y. Yuan, C. Wang, K. Lei, H. Li, F. Li, J. Chen, Sodium-ion hybrid capacitor of high power and energy density, ACS Cent. Sci. 4 (2018) 1261-1265.

9. Y. He, A. Liang, D. Zhu, M. Hu, L. Xu, S. Chao, W. Zhou, Y. Wu, J. Xu, F. Zhao, Organic-inorganic hybrid electrode engineering for high-performance asymmetric supercapacitor based on WO3-CeO2 nanowires with oxygen vacancies, Appl. Surf. Sci. 573 (2022) 151624.
